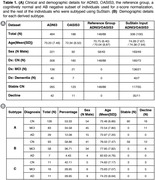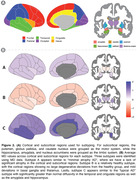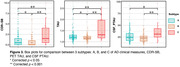# Data driven subtyping of individuals on the Alzheimer’s continuum using cortical microstructure from diffusion MRI

**DOI:** 10.1002/alz.089948

**Published:** 2025-01-09

**Authors:** Shayan Javid, Alyssa H Zhu, Iyad Ba Gari, Neda Jahanshad, Talia M Nir

**Affiliations:** ^1^ Imaging Genetics Center, Mark and Mary Stevens Neuroimaging & Informatics Institute, University of Southern California, Marina del Rey, CA USA

## Abstract

**Background:**

Alzheimer’s disease (AD)‐related pathologic changes in the brain begin years before MRI‐detected atrophy and the onset of cognitive decline. Diffusion MRI (dMRI) can quantify microstructural alterations in brain tissue, and may be more sensitive to pathologic processes than standard MRI volumetric biomarkers. As AD is a heterogeneous disease, previous studies have used post‐mortem, structural MRI, and PET measures to subtype individuals by areas of the brain affected, identifying distinct patterns of degeneration and decline. Subtyping individuals using non‐invasive dMRI microstructural alterations, which likely occur before volumetric atrophy, may provide complementary assessments of possible trajectories of decline. We pool two datasets to subtype individuals using a data‐driven approach, and determine whether there are subtype differences in cognitive impairment, and more invasively derived metrics (PET and CSF derived assessments of beta‐amyloid (Aβ) plaques or tau tangles).

**Method:**

T1w and dMRI data were processed for 484 ADNI3 and 188 OASIS3 participants (Figure 1A). FreeSurfer was used to segment cortical lobes and subcortical structures; thickness or average MD was calculated in each. T1w and dMRI measures were harmonized across imaging protocols using ComBat. We used SuStaIn (Young 2018) to subtype individuals based on regional dMRI‐derived mean diffusivity (MD) and separately, based on cortical thickness and subcortical volume. We determined how derived subtypes relate to PET or CSF AB and tau levels, and cognitive scores using mixed effects regressions, covarying for age, sex, MRI scanning protocol, and diagnosis.

**Result:**

SuStaIn identified three subtypes for both T1w and MD data. When analyzing MD‐derived subtypes, significant pairwise differences between all subtypes were found for cognitive scores and tau PET. Progression and CSF tau was significant when comparing typical AD with the less altered MD subtypes. T1w‐derived subtypes differed significantly in cognitive scores but not tau measures. There were no significant associations with Aβ.

**Conclusion:**

SuStaIn identified T1w and MD subtypes that reflect previously suggested subtypes, such as typical AD and minimal atrophy AD. Our findings comparing dMRI‐derived subtypes indicate that MD may be sensitive to tau aggregation. Future studies will evaluate staging, include a finer cortical parcellation, and data from additional studies to ensure generalizability of findings.